# Experimental model for the description of the behaviour of a 9-mm projectile at a target

**DOI:** 10.1080/20961790.2019.1697078

**Published:** 2020-04-09

**Authors:** Patricia Morales-Vega, Daniel Esteban Jaramillo-Arango, Jaime Alberto Osorio-Velez, David Noreña-Blandón

**Affiliations:** Instituto de Física, Universidad de Antioquia, Medellín, Colombia

**Keywords:** Forensic sciences, forensic ballistics, firearm, gyroscopic motion, Euler equations, nutation, precession, projectile motion

## Abstract

Analysis of crime scenes involving single-fire-gun projectiles requires the determination of the direction of arrival of a projectile at the target and other factors to reconstruct events. The movement of a projectile can be analyzed by applying Euler’s equations to a solid symmetrical rigid body. The present work starts from a Newtonian reformulation of these equations to show that, in the presence of a gravitational field, the system can be expressed with a complex variable nonlinear equation, where the inclusion of small nutation variables allows us to find possible solutions. As a particular case, we analyzed the movement of a 9-mm projectile fired from distances greater than 1 m to demonstrate that the direction of arrival of the projectile at the target cannot be traced by a stick placed in the target hole, as is usually performed in crime investigations. A series of shots were fired from distances varying between 1 m and 7 m. Impact data were recorded on Riemann planes of projection for the description of nutation and precession motions, allowing the observation of the motion dynamics of the projectile. We show that the direction of arrival at the target can be determined approximately from the analysis of the nutation and precession curves through Riemann planes of projection. The results presented in this work will allow more accurate judgements to be made in judicial investigations.

## Introduction

It is a fundamental matter to determine the characteristics of shots fired in scenarios where firearms have been used. The correct determination of these characteristics allows a more accurate investigation that identifies a possible location from which shots have been fired and the firearm used. The estimation of the bullet trajectory traditionally starts by placing a fibreglass rod into the bullet hole of the target. Although this procedure is inaccurate, it is, at least, possible to infer geometrical parameters that can be used in rebuilding the kinematics (i.e. both trajectory and tumbling movement) of a projectile.

Euler equations or their Newtonian, Lagrangian and Hamiltonian reformulations are usually employed to describe the movement of a symmetric solid body, namely a bullet. Previous work [1] showed that in the presence of dissipative forces and a gravitational field, such equations can be reduced to a nonlinear equation over the components of the Riemann stereoscopic projection of the main vector on a plane tangent to the tip of the projectile in the direction of the gravitational field. Under the approximation of small-amplitude nutations, a solution to the equation can be found and modified to include the movement of the projectile [1].

Projectile motion can be explained theoretically in an approximate way using the Lagrangian formulation of Newtonian mechanics. However, this explanation remains imprecise and incomplete. The projectile motion exhibits a more intuitive physical richness in that it is not limited to elliptical motion but also presents nutation and precession movements. Such motions of nutation and precession are not well described by less-elaborated formulations of mechanics. The study of such nutation and precession is important in that it allows us to describe the precise path followed by the projectile and the initial conditions of the shooting.

There are no reports of the described experimentation or quantification and qualification of ballistic elements for different-calibre projectiles because the experiments are expensive and difficult to process with the authorities. The police and army are the only parties allowed to possess high-calibre weapons and ammunition. It is thus difficult for a civilian or academic institution to conduct experiments even though experimental techniques will be easily reproducible if bureaucratic obstacles are overcome.

The aim of the present work is to show that the gyroscopic motion of a bullet fired by a firearm directly shapes the figure of the target hole, contradicting conclusions that might be made about the trajectory on the basis of the aforementioned rod procedure. Therefore, another degree of freedom is added to the problem of the trajectory that must be resolved in subsequent investigations.

We compare the description of the motion of a commercial gyroscope that exhibits epicyclic movement with the same motion of a 9-mm bullet in flight and over Riemann planes of projection (RPPs). To this end, we examine the incidence of projectiles into targets using the data of experiments and ballistic tests. Such data reveal the nutation and precession movements, which are generally observed in computational simulations but not experimentally.

The present paper is arranged as follows. The second section presents the methodology implemented and describes the experiment and its theoretical framework. The third section summarizes our main findings. The fourth section discusses the results and presents conclusions.

## Materials and methods

Euler equations are used to establish the relationship among the distance, direction of firing by the firearm and the projectile entrance footprint. This paper presents an experiment that, on the basis of the footprints of projectiles on RPPs, reveals that the initial firing conditions and the trajectory may not coincide with the projectile entry angles in the target for any firing distance.

### Description of the experiment

Twenty-five sheets of straw cardboard were used as RPPs. The sheets had low impact resistance so to smoothly destabilize the projectile and produce a clear footprint. The RPPs each had an area of 17.5 cm by 25 cm and a thickness of 0.095 mm. The RPPs were placed at intervals of 25 cm. There was a sequence of 25 planes per round of firing. In this way, a clear bullet footprint was registered for each plane, allowing the extraction of all required information. The shooter was initially located 1 m from the first RPP in the sequence, and the distance between the shooter and the first RPP was then increased in intervals of 1 m to a final distance of 7 m.

**Figure 1.**
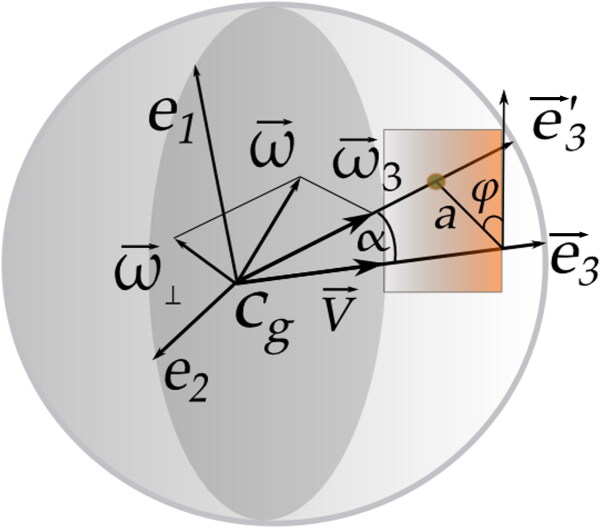
Vectoral diagram showing the attack vector and its relationship with angles and angular velocities.

Angles and longitudes were directly measured using digital calibrators for all footprints, giving the plane of incidence of the projectile or RPP. Figure 1 presents measurements of the size of the footprint made by the projectile and the angles that allow the determination of the quantities associated with precession and nutation from the projectile profile.

### Equations of motion and parametrization for the projectile case

**Figure 2.**
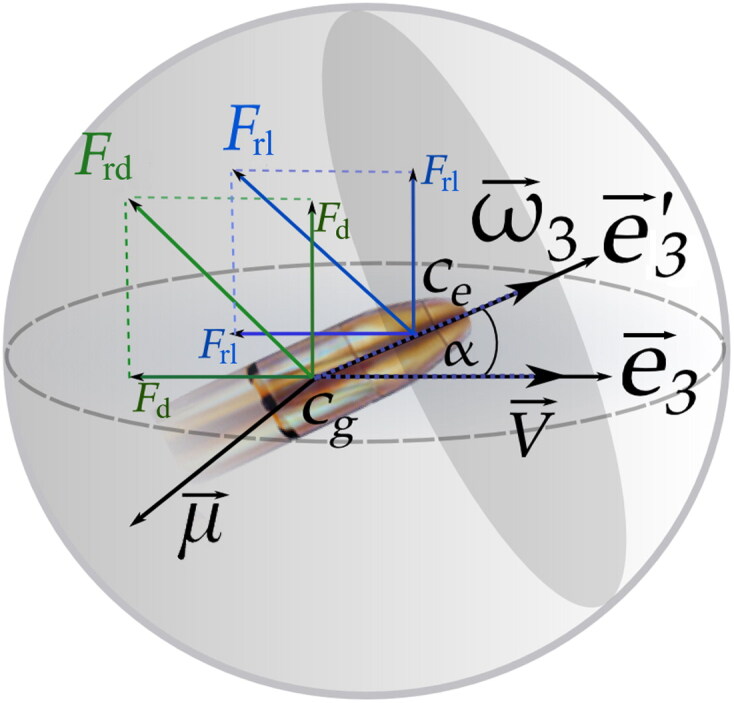
Diagram of the principal forces acting on a projectile. $F_{l}$ is the lifting force, $F_{d}$ is the aerodynamic friction, $\vec{\omega }$_3_ is the rotational velocity component along the $e_{3}^{\prime }$ axis, $\vec{µ}$ is the aerodynamic torque, and α is the angle of attack. $C_{g}$ and $C_{e}$ are the centres of gravity and lift, respectively.

The complete motion of the projectile is determined by the forces and torques acting on the projectile (Figure 2). All these effects determine the conditions of motion of the projectile’s centre of gravity.

Inside a gun, the advance of the projectile is guided by the bore of the barrel. The bore imprints a rotation motion on the projectile using several helical guiding fields with left and right inclinations. The bullet faces environmental perturbations when it leaves the barrel muzzle. The bullet continues to travel with an attack vector, resulting in an aerodynamic re-elevation phenomenon that vanishes inside the weapon. Such perturbations are due to the interactions of gases in the muzzle and the local atmospheric air over a short time interval, which is known as the ballistic wind phenomenon [2].

As the projectile travels through air, the motion of the centre of gravity is determined by Newton’s second law (Equation (2.1)):

(2.1) $$m\dot{v}=F_{W}+F_{d}+F_{l}+F_{M}.$$

Air resistance generates forces and moments that act on the projectile and can be determined experimentally using aerodynamic coefficients. These coefficients are not necessarily constant and can be expressed as complex polynomial functions, which in turn depend on the projectile attack angle and variables dependent on the Mach number (0.94 for 9-mm ammunition). Aerodynamic coefficients are usually evaluated in subsonic and supersonic tunnels depending on the case [3].

To better understand the characteristics of a flying projectile, additional concepts, such as those determining docility and stability, must be considered because these characteristics are what make each flying projectile unique [3].

The projectile stability depends on the rotation speed, which must be adjusted such that the effects of the medium, such as air and gravity, do not turn the direction of the tangent axis ${\hat{e}}^{\prime }$ away from the trajectory. This is how damped gyroscopic motion is generated. Despite environmental interactions, the deviation of the tip of the projectile is negligible if the docility is small, which we can ensure. In such a case, the docility is due only to nutation and precession. The projectile is said to be stable if it does not turn under environmental effects. Docility is defined as

(2.2) $$\delta r\frac{2g}{v}\sqrt{\frac{I_{2}S_{s}}{\mu }}.$$

### Attack vector and essential stability of the projectile

A projectile moving around its centre of mass has a rotational velocity $\vec{\omega }$ with two components, i.e. the rotational velocity around the principal rotation axis, ${\vec{\omega }}_{3},$ due to the projectile’s own rotation and the transversal or perpendicular component that is in the plane formed by ${\hat{e}}_{1}$ and ${\hat{e}}_{2},$ denoted ${\vec{\omega }}_{⊥}={\vec{\omega }}_{1}+{\vec{\omega }}_{2}$ (Figure 1).

When a rigid body rotates around a principal inertial axis, the angular momentum $\vec{L}$ is parallel to the angular velocity $\vec{\omega }$ and is always directed along the rotation axis. For a symmetric rigid body with $I_{1}=I_{2},$ the angular momentum can be written as the sum of longitudinal and transversal angular momenta:

(2.3) $$\;\vec{L}=I_{3}{\vec{\omega }}_{3}+I_{1}{\vec{\omega }}_{⊥}.$$

The projection plane is perpendicular to the ${\vec{e}}_{3}^{\prime }$ axis while the principal axis ${\vec{e}}_{3}^{\prime }$ of the projectile describes the motion of the projectile in the RPP. The attack vector, $\vec{a},$ is located on the RPP and corresponds to a vector radius that extends from the cut of the ${\vec{e}}_{3}$ axis with the RPP to the point of intersection of ${\vec{e}}_{3}$ with the same plane.

Starting with the Euler equation, it is possible to determine the exact solution in particular cases under certain approximations. Here, the procedure is applied to the case of a projectile [4]:

(2.4) $$\vec{\tau }=\dot{I}\vec{\omega }+I\dot{\vec{\omega }}+\vec{\Omega }\times I\vec{\omega }.$$

A differential is obtained using Equation (2.3) for small angles (i.e. cos *θ ≈* 1) and letting $a=Ae^{\text{st}}$ [1]:

(2.5) $$I_{2}\dot{a}-iL_{3}\dot{a}-\mu a=0.$$

The solution is

(2.6) $$S_{\pm }=\frac{iI_{3}\omega_{3}\pm \sqrt{4I_{2}\mu -I_{3}^{2}\omega_{3}^{2}}}{2I_{2}}.$$

Two types of solution are obtained for the above equation in the case of a projectile.

- If the equation discriminant is positive, a complex solution for S with a positive real part and null imaginary part is obtained. This corresponds to a function that grows indefinitely with time. In conclusion, as the moment increases, the projectile rotates through *π* radian, resulting in the tip moving backwards and the base moving in the direction of motion. The motion thus becomes unstable.
- If the equation discriminant is negative, then two imaginary roots are obtained and the solution to Equation (2.5) becomes
  (2.7) $$S_{\pm }=ik\left(1\pm d\right).$$
  where
  $$k=L_{3}/(2I_{2})\text{ and }d=\sqrt{1-4\mu I_{2}/L_{2}^{3}}.$$

The more general solution to Equation (2.5) is

(2.8) $$a=a_{p}e^{i\left(\omega_{p}t+\phi_{p}\right)}+a_{n}e^{i\left(\omega_{n}t+\phi_{n}\right)},$$

where the first term represents a rotating vector having amplitude $a_{p}$ and angle $\omega_{p}t+\phi_{p}$ that varies with time while it rotates around the centre of gravity with slow circular motion at what is called the precession angular velocity. The second term represents another rotating vector of amplitude $a_{n}$ and angle $\omega_{n}t+\phi_{n},$ which is in the extreme of the $a_{p}$ vector, with a faster twist, presenting a circular motion with what is called the nutation angular frequency. There is more than one lap of the second vector for each lap of the first vector. The vector obtained by summing the first and second vectors is therefore the attack vector $\vec{a},$ which rotates through an angle ϕ as shown in Figure 1. $\vec{a},$ therefore, determines the resulting motion of the projectile tip and the epicyclic trajectory on the RPP. The angle ϕ does not coincide with the precession angle, except when the nutation angle vanishes.

The solutions of $\omega_{p}$ and $\omega_{n}$ are complex solutions of the precession frequencies $\omega_{p}\;$ (Equation (2.9)) and nutation (Equation (2.10)), with the condition $\omega_{p}\to \omega_{n}-\omega_{p}$ where $\omega_{p}<\omega_{n}.$ The equation of motion of the gyroscopic movement is thus given by Equation (2.5), for which the constants $a_{p}$ and $a_{n}$ correspond to complex numbers that are determined from the initial conditions

(2.9) $$\omega_{p}=k\left(1-d\right),$$

(2.10) $$\omega_{n}=k(1+d).$$

**Figure 3.**
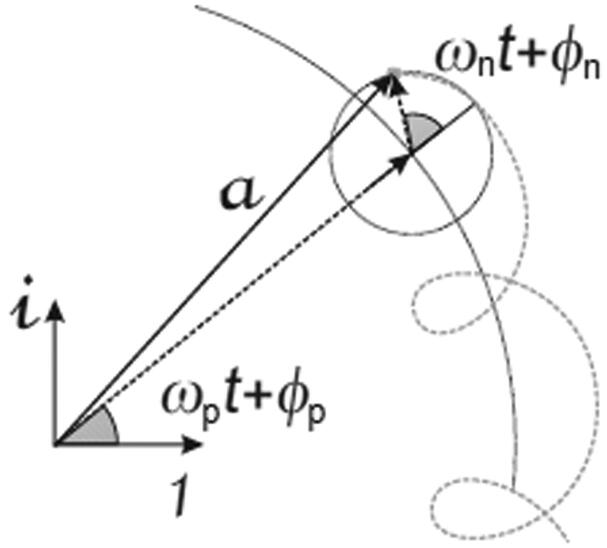
Graphical interpretation of the gyroscope solution.

As a consequence, stability of the projectile demands that the discriminant of Equation (2.5) must be negative; i.e. $4I_{2}\mu -I_{3}^{2}\omega_{3}^{2}<0$ or equivalently

(2.11) $$S_{s}=\frac{I_{3}^{2}}{4I_{2}\mu }>1.$$

where $S_{s}$ is referred to below as the gyroscopic stability coefficient. It is concluded from the above that the projectile is stable if $S_{s}=I_{3}^{2}\omega_{3}^{2}/\left(4I_{2}\mu \right).$ This is to say, a projectile is stable if its stability coefficient is greater than 1, as will be shown experimentally. As the projectile has gyroscopic motion, the precession and nutation motions can be calculated experimentally. Likewise, the value of the angle that corresponds to the rotating vectors, having amplitudes $a_{p}$ and $a_{n},$ respectively given by Equations (2.9) and (2.10) for the limit $\theta_{0}\to 0.$ The geometric interpretation of the motion described by Equations (2.9) and (2.10) allows the plotting of the projectile tip motion (Figure 3). In the case of the projectile, epicyclic motion appears only with apices facing inwards. It is thus affirmed that the projectile tip describes a gyroscopic motion, represented by a flat curve in the RPPs (Figure 3), with apices facing inwards, tangent to a sphere that has as its origin at the projectile centre of gravity and a radius that is the distance from the centre of gravity to the tip. (The centre of gravity is located at one-third of the projectile longitude from the base of the projectile, as determined by the conical symmetry.)

## Results

On the basis of previously developed theoretical models [1], we obtained a set of results in gyroscope and projectile experiments. In the case of a projectile that we are dealing with here, an experimental assembly is made for different parameters and different shooting distances, which are analyzed in the following manner.

- The angles of precession and nutation are plotted against the shooting distance in meters.
- Precession and nutation frequencies are determined from those plots.
- With information on ballistics taken from the literature, the projectile rotation frequency and aerodynamic coefficient are obtained for comparison of the theoretical torque with the experimental torque [2].
- The number of loops per lap, given by $N=\omega_{n}/\omega_{p},$ is calculated from the data in Table 1, which were experimentally obtained for the 9-mm projectile. This definition is fulfilled by both the gyroscope and projectile and the angular frequency between the fast and slow vectors. The time between two amplitude maxima is given by $P=2\pi /\omega_{n},$ while the period for the slow vector is $P_{1}=2\pi /\omega_{p}.$ From the experimental data, we calculate $N=2d/\left(1-d\right)$ only for the projectile [3].

**Table 1.** Dynamic quantities calculated from measurements of the target.

Shooting distance (m)	$S_{s}$ stability	$\omega_{p\;}\left(s^{-1}\right)$	$\omega_{n\;}\left(s^{-1}\right)$	*P* (s)	μ (Nm)
1	1.20	572.389	2940.798	0.0024	0.582
2	1.29	964.135	2635.205	0.0023	0.526
3	2.39	407.817	3191.523	0.0024	0.458
4	1.99	298.031	3301.309	0.0022	0.621
5	2.05	553.498	3045.842	0.0026	0.699
6	1.77	772.265	2827.075	0.0029	0.602
7	1.91	638.573	2960.767	0.0028	0.557

**Figure 4.**
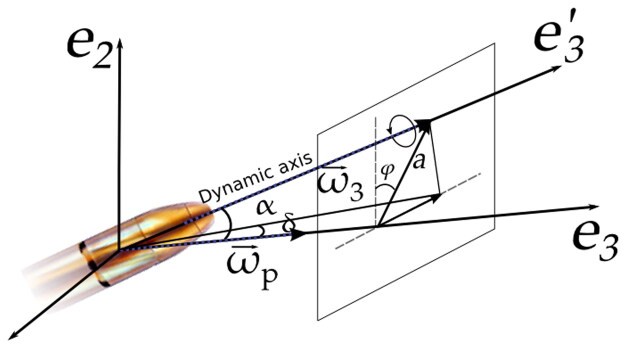
Dynamical axis of rotation and angles φ, α and δ for the 9-mm projectile in flight.

The stability coefficient given by Equation (2.11) allows the determination of the docility given by Equation (2.2). From the calculation of this parameter and using ballistic data obtained from the technical specifications of the gun and projectile and measurements of the RPPs, the value of d is estimated as $d=\sqrt{1-1/S_{s}}.$ The determination of the precession angle θ yields $\omega =I_{3}\omega /\left(2I_{2}\right),$ $\mu =I_{3}^{2}\omega^{2}/\left(4I_{2}S_{s}\right).$ The step of the gun striation $n=2\pi v_{0}/\omega$ according to Equation (2.4) (Figure 4).

**Figure 5.**
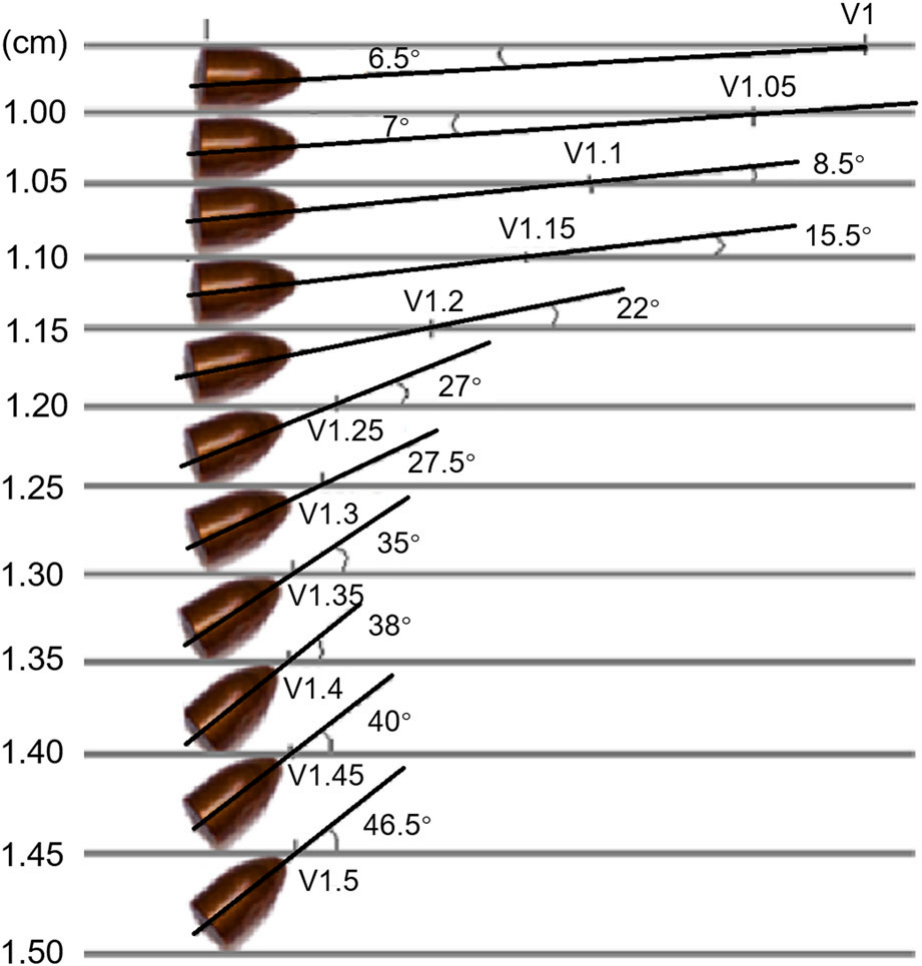
Graphical determination of the rotation angle α according to the tangent to the trajectory.

**Figure 6.**
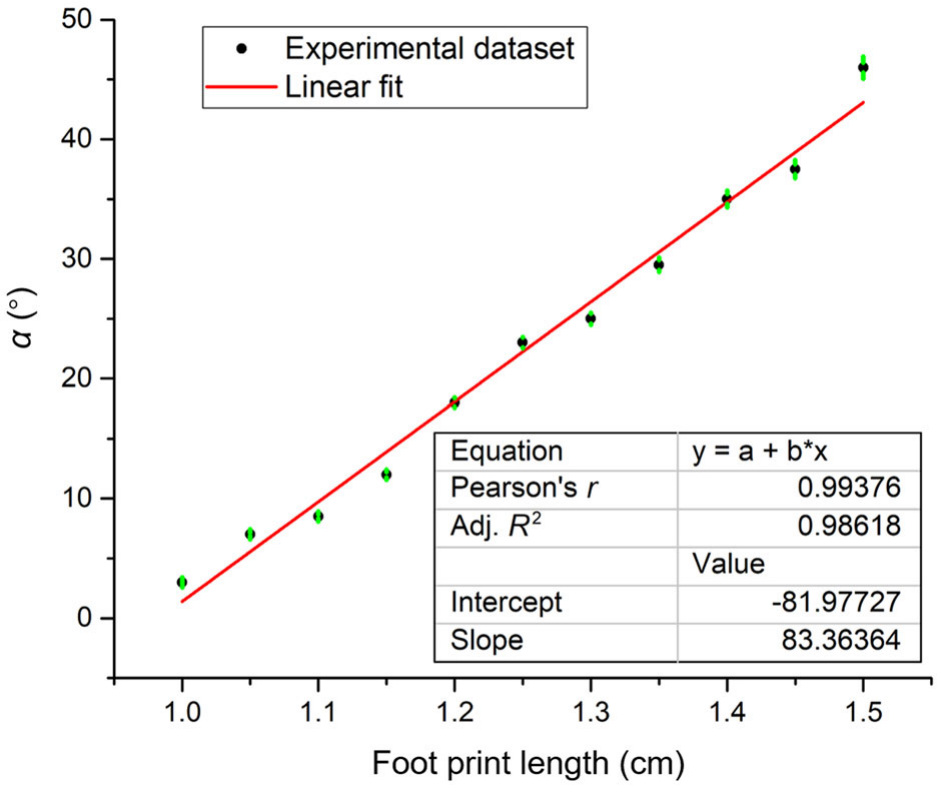
Nutation angle as a function of the footprint length in the target.

To obtain the information needed to perform the calculations using the aforementioned equations, it is necessary to determine the attack angles of the footprints left by the projectile on the RPPs, the lengths of bullet holes, and the distance between tangential points for different angles. With the data obtained for the footprint direction and longitude on the RPPs, the relationship between the longitude and direction is established to determine the curve with the profile of a real projectile and the separation of tangential points is measured for different angles as shown in Figure 5. With this information, we fit a curve to the data (Figure 6), where the variation in the projectile footprint with a variation in the attack angle is seen, resulting in the explicit relation expressed by Equation (3.1). The curve in the figure has a linear behaviour with the footprint data given in millimetres. The relation is

(3.1) α(L) = 8.34L −(81.97±0.01),

where *L* is the footprint length. The coefficient of determination for this case affirms that Equation (3.1) explains the variation in the nutation angle as a function of the footprint length in the RPP with a 99% level of confidence (Figures 5 and 6). Using Equation (3.1), the relation between the projectile footprint measured in the RPP and the shooting distance is analyzed below.

In Table 1, *P* is the period between two consecutive maxima while the torque μ is calculated with the stability coefficient and the factor *d*, whereas $L_{3}$ is calculated with $\omega_{p}$ (percentage error of 5%).

The magnitudes of the precession and nutation frequencies are adjusted to expected values with a coefficient of determination that on average has an 81% level of confidence, as shown by the aerodynamic results obtained from the general test and from the plots. The average value is obtained from the analysis of 150 targets studied.

**Figure 7.**
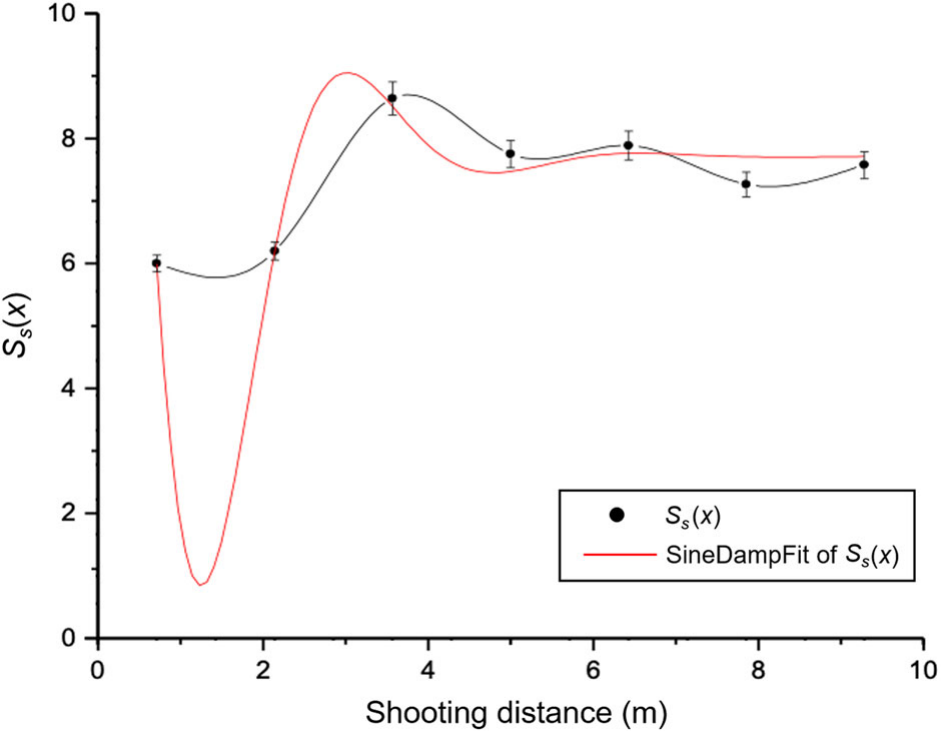
Stability factor as a function of the shooting distance (9-mm calibre ammunition).

The deviations for non-controlled situations in the quantitative analysis of the data recorded for the target footprints relative to expected or theoretical values are a consequence of environmental conditions, such as the wind effect, and the time between shots. This prevented the required stability of RPPs from being fully achieved. Nevertheless, it is possible to appreciate the damping of the projectile motion from the relation of the nutation angle against the shooting distance as shown in Figure 7.

To evaluate the results for the precession frequency and stability, we considered solutions (2.9) and (2.10) to the differential Equation (2.5) with parameter values fitted to the trajectory shown in Figure 7. From the fitting curve, the gyroscopic stability factor for the barrel muzzle is obtained as $S_{s}\left(x=0\right)=-4.07$ for the 9-mm calibre projectile with an 89% level of confidence. This result is an approximation of the dynamical stability under real conditions, as seen in Figure 7, where the stability factor is analyzed with respect to the shooting distance.

Further stability analysis is conducted for a distance ranging between 1 m and 7 m. The red curve in Figure 7 has a trend similar to the theoretical trend. Table 1 shows that the projectile is generally stable, with the stability coefficient fluctuating between 1.33 and 2.50, which is accepted for low-calibre ammunition [5].

It is appropriate at this point to comment on the experimental results relating to Table 1, where the calculated dynamical quantities are given. The stability factor fits the theoretical predictions, as the stability is greater than unity for subsonic projectiles; i.e. has values between 1.20 and 2.39. Moreover, Figure 7 shows that the factor $S_{s}$ is proportional to the square of the rotational speed of the projectile, corresponding to the behaviour of a projectile stabilized by rotation due to striation of the weapon. This result agrees with theory that establishes that projectiles without fins are generally spin stable. However, the stability factor increases for low-calibre projectiles, explaining the jump of the curve in Figure 7. The results in Table 1 thus suggest that the stability factor has a certain level at 3 m and tends to oscillate with decreasing amplitude from this distance until it approaches a constant value with increasing distance, because there it is still a nutation frequency, which vanishes at a distance of approximately 200 m [3].

The following presents relations obtained in the experiments. First, experimental Equation (3.2) describes the relation of stability versus the shooting distance, where the values 1.99 and 2.13, respectively, correspond to values of *x* that determine the amplitudes of oscillation, showing that the curve fits the theoretical parameters [5] with an 89% level of confidence. It is worth clarifying that there is dynamical stability when $S_{s}>1\;$ (theoretical value $S_{s}>1.33$) while the graphical interpretation of the aerodynamic jump between 1.00 and 2.50 m suggests that this jump is due to the chaotic nature of the motion at the beginning of the trajectory (i.e. the ballistic wind). Therefore, from (3.2), with *x* = 0, we obtain $S_{s}=-4.07,$ which is in the expected range. After this event, the projectile recovers its sinusoidal motion around the centre of mass and must continue tangent to the trajectory.

The relation of stability versus the shooting distance is

(3.2) $$S_{s}\left(x\right)=1.96+57.44e^{-x/0.69}\;\text{sin}\;\left[\pi \left(\frac{x-2.08}{1.1}\right)\right]$$

**Figure 8.**
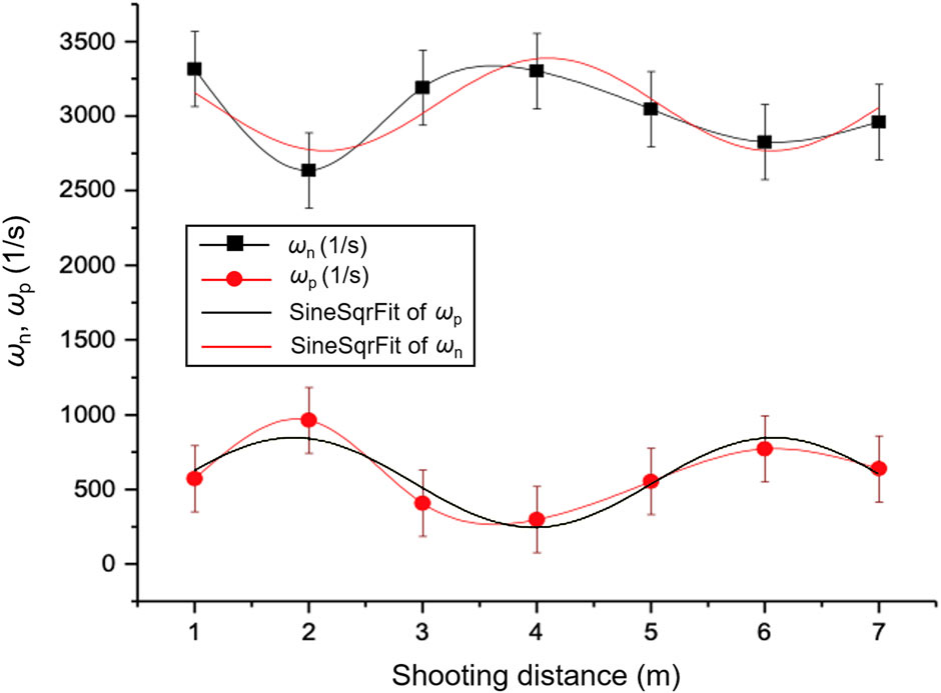
Nutation frequency (upper series) and precession frequency (lower series) as functions of the shooting distance.

Second, Figure 8 presents the adjusted curves for the precession and nutation frequencies as functions of the shooting distance. These angular motions confirm that the centre of mass tends to follow the precession and nutation movements that the projectile tip describes, as seen in Figure 1. The precession reaches a minimum when the nutation reaches a maximum and *vice versa* as the shooting distance with respect to the RPP varies. It is considered for this experiment that the tip of the flying projectile is in a state of low nutation that approximates it in a small ϕ to the horizontal plane. The relations of the angular frequencies of nutation and precession with the shooting distance in Figure 8 are respectively,

(3.3) $$\omega_{n}=3045.693+303.244\;\text{sin}\;\left[\pi \left(\frac{x-1.452}{2.152}\right)\right]$$

(3.4) $$\omega_{p}=546.022+300.652\;\text{sin}\;\left[\pi \left(\frac{x-0.816}{2.103}\right)\right]$$

A value of 3037.38 s*^−^*^1^ is read for the angular frequency of nutation in contrast with the value of 554.27 s*^−^*^1^ for precession. The adjusted value for the nutation frequency is 3054.01 s*^−^*^1^, in contrast with a value of 537.77 s*^−^*^1^ for the precession frequency with an 81% level of confidence. These results show clearly that the slow circular motion corresponds to the angular frequency of precession and the fast circular motion corresponds to the angular frequency of nutation as is predicted by theory [5]. Additionally, Figure 8 shows a dependence of these frequencies on the shooting distance due to stability.

**Figure 9.**
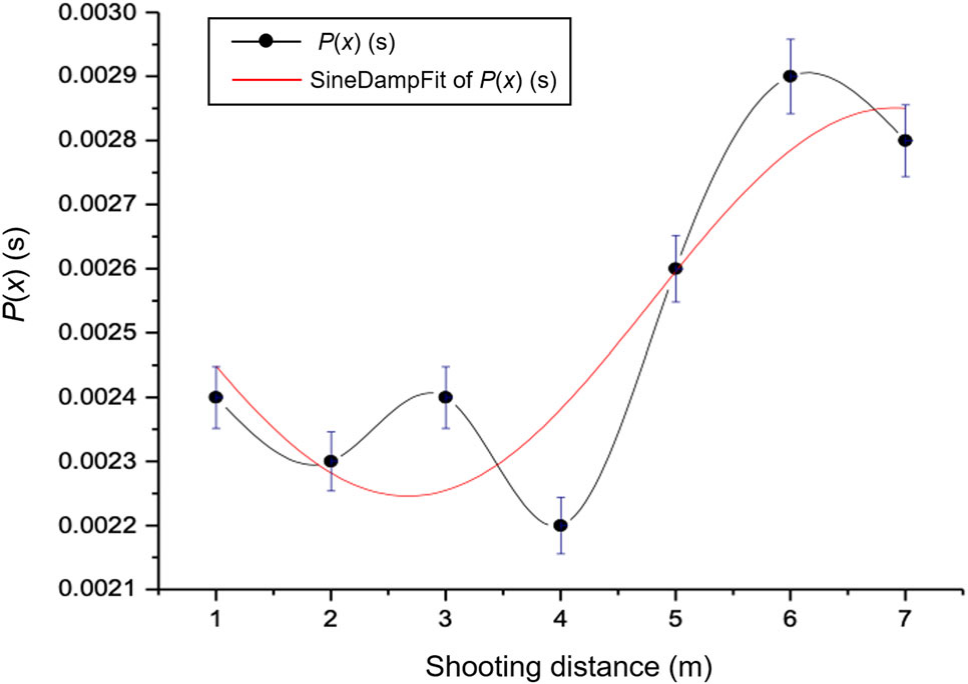
Oscillation period as a function of the shooting distance.

Third, Figure 9 and Table 1 show that the oscillation period of the projectile remains between 0.0022 and 0.0029 s as the shooting distance varies. The periods for shooting distances between 1 and 3 m are between 0.0024 and 0.0023 s, indicating that the period is at an average amplitude of the curve and therefore showing stability. After 4 m, the curve destabilizes, suggesting that external factors affect the experimental results. The relation between the oscillation period and shooting distance is

(3.5) $$P\left(x\right)=0.0023+6.3340\mathrm{si}n^{2}\left[\pi \left(\frac{x-2.6600}{8.8190}\right)\right]$$

The oscillation period has a minimum value of 0.00225 s and a maximum value of 0.00234 s, with a 70% level of confidence according to the fitting curve.

**Figure 10.**
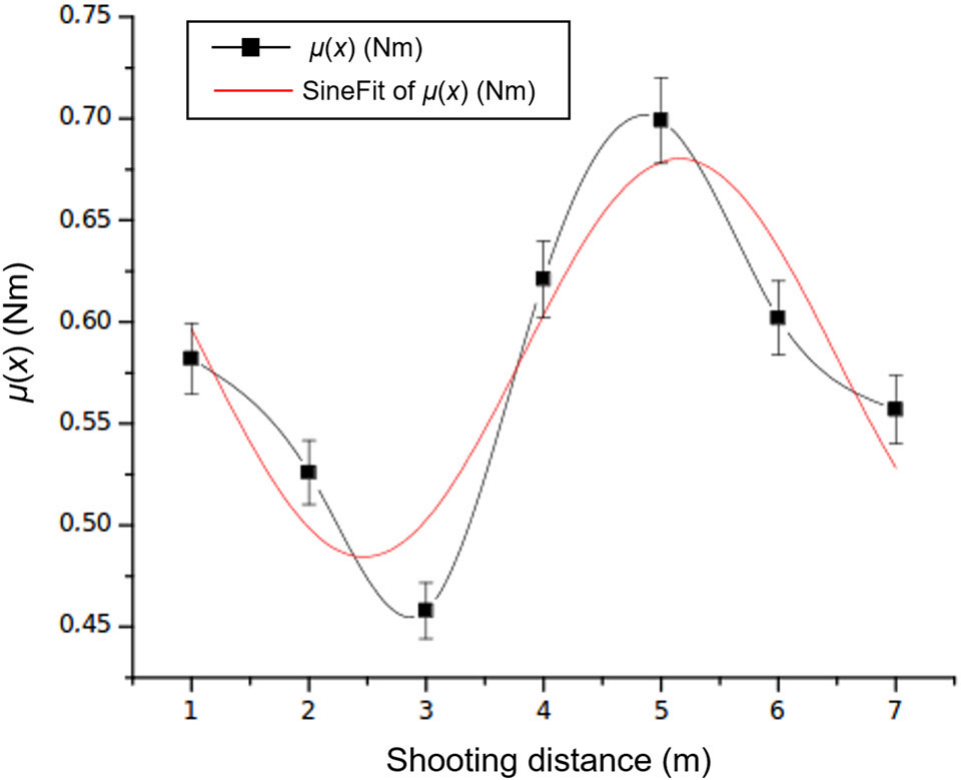
Torque as a function of the shooting distance.

Finally, the relationship found between the moment and shooting distance is shown in Figure 10. The proposed model expressed by Equation (3.6) explains with a 68% level of confidence the variation in the torque as a function of the shooting distance. The experimental fitted value for the moment is 0.497 Nm, which is comparable to the theoretical value of 0.473 Nm [2]. The model thus explains the theoretical and experimental behaviours with an error of 5.07% approximately. The proposed model is

(3.6) $$\mu \left(x\right)=0.484+0.196s{\mathrm{in}}^{2}\left[\pi \left(\frac{x-2.470}{5.374}\right)\right]$$

It is interesting to examine the experimental results of the torque–stability relationship, highlighting that the variables are counterposed and that the projectile is docile. $S_{s}$ is, therefore, the solution to differential Equation (2.5). We thus confirm the exterior ballistics theorem [3] and the validity of the experiment.

## Conclusion

Through Riemann projection, the vector Euler equation was reduced to a nonlinear differential equation of a single complex variable. This equation was solved implementing the constant module approximation [1]. The obtained solution is the sum of two vectors rotating on a complex plane. The vector having the greatest magnitude rotates at lower angular velocity while the shorter vector rotates at higher angular velocity. The speed of precession corresponds to the speed of rotation of the vector of greater magnitude, while the speed of nutation corresponds to the difference in speed between the two vectors.

Analysis of the results of experiments designed for a projectile revealed that, on the basis of the theory and experimental data obtained from the projectile’s footprint at the RPP (e.g. the length and shape), for distances greater than 1 m, the same obliquity characteristics are found regardless of the distance or direction of the shot.

Analysis of the curves of the frequencies of nutation and precession described the arrival of the projectile at the target and determined the trajectory through the target, whether it be ascending, descending, or level, as shown in Figure 8. The proposed methodology contrasts with methods used commonly in certain areas; i.e. judicial procedures that conclude the origin of the shot from the direction provided by the contusion are *a priori* results.

Physical and mathematical descriptions of the projectile movement, in general terms, present certain difficulties, because motion descriptions correspond to complex differential equations whose solution is determined by environmental conditions. The simplified solution of these equations depends on gyroscopic movement, the phenomenon of drift and re-elevation.

The nutation angle and precession in RPPs were measured to quantify the gyroscopic stability in the aerodynamic model of a 9-mm calibre projectile. Experimental data were plotted with respect to the shooting distance, with coefficients of determination ranging between 0.68 and 0.87. These values indicate the explanatory capacity of the fit corresponding to Equation (3.2) and characterize a projectile at a target.

The experimental data and the calculations made for the 9-mm calibre projectile reveal that the number of rotations per nutation cycle is a linear function of the ratio between the precession and nutation velocities and the average angle of inclination of the projectile axis. This finding agrees with the literature on external ballistics and the results of previous work [1].

When single-projectile impacts are presented, it is necessary to determine the direction of arrival and other possible factors to reconstruct events for the purposes of judicial investigation. The nutation and precession movements are not observable empirically from the projectile footprint *because* these movements cannot be registered using high-speed cameras or computer software. The analysis conducted in this work is applicable to the relation of the shooting distance versus the Euler angle.

Meanwhile, evidence relating to the contusion line, the distribution of embedded powder grains and black smoke are criteria only used for detonations made at close distance. At larger distances, the direction of entry into the target is determined by the shape that suggests the obliquity of the entrance of the projectile to the surface of impact. These criteria must be re-evaluated because distances greater than 1 m have the same characteristics of obliquity in the contusion because the phenomena of nutation and precession generate the same shapes of entry in the target for different distances and directions (Figure 8).

## References

[1] Morales P, Jaramillo DE. Experimental aspects of the gyroscope movement. Rev Mexi Fis. 2016;62:44–50.

[2] Sellier K, Kneubuehl B. Wound ballistics and the scientific background. D-53111. Amsterdam (Netherlands): Elsevier; 1994.

[3] Cucharelo FP. Balistica exterior. Madrid (Spain): Ministerio de Defensa; 1994.

[4] Goldstein H, Poole C, Safko J. Classical mechanics. 3rd ed. Boston (MA): Addison-Wesley; 2002. p. 184–188.

[5] McCoy RL. Modern exterior ballistics. Atglen (PA): Schiffer; 1999.

